# An individually-tailored multifactorial intervention program for older fallers in a middle-income developing country: Malaysian Falls Assessment and Intervention Trial (MyFAIT)

**DOI:** 10.1186/1471-2318-14-78

**Published:** 2014-06-21

**Authors:** Pey June Tan, Ee Ming Khoo, Karuthan Chinna, Keith D Hill, Phillip JH Poi, Maw Pin Tan

**Affiliations:** 1 Ageing and Age-Associated Disorders Research Group, Health and Translational Medicine Cluster, University of Malaya, Kuala Lumpur, Malaysia; 2 Department of Primary Care Medicine, University of Malaya Primary Care Research Group (UMPCRG), Faculty of Medicine, University of Malaya, Kuala Lumpur, Malaysia; 3 Department of Social and Preventive Medicine, Faculty of Medicine, University of Malaya, Kuala Lumpur, Malaysia; 4 School of Physiotherapy and Exercise Science, Faculty of Health Sciences, Curtin University, Perth, Western Australia, Australia; 5 Division of Geriatric Medicine, Department of Medicine, Faculty of Medicine, University of Malaya, Kuala Lumpur, Malaysia; 6 Health Promotion Division, National Ageing Research Institute, Parkville, Melbourne, Victoria, Australia

**Keywords:** Accidental falls, Aged, Asians, Randomized controlled trial, Fear of falling, Quality of life

## Abstract

**Background:**

In line with a rapidly ageing global population, the rise in the frequency of falls will lead to increased healthcare and social care costs. This study will be one of the few randomized controlled trials evaluating a multifaceted falls intervention in a low-middle income, culturally-diverse older Asian community. The primary objective of our paper is to evaluate whether individually tailored multifactorial interventions will successfully reduce the number of falls among older adults.

**Methods:**

Three hundred community-dwelling older Malaysian adults with a history of (i) two or more falls, or (ii) one injurious fall in the past 12 months will be recruited. Baseline assessment will include cardiovascular, frailty, fracture risk, psychological factors, gait and balance, activities of daily living and visual assessments. Fallers will be randomized into 2 groups: to receive tailored multifactorial interventions (intervention group); or given lifestyle advice with continued conventional care (control group). Multifactorial interventions will target 6 specific risk factors. All participants will be re-assessed after 12 months. The primary outcome measure will be fall recurrence, measured with monthly falls diaries. Secondary outcomes include falls risk factors; and psychological measures including fear of falling, and quality of life.

**Discussion:**

Previous studies evaluating multifactorial interventions in falls have reported variable outcomes. Given likely cultural, personal, lifestyle and health service differences in Asian countries, it is vital that individually-tailored multifaceted interventions are evaluated in an Asian population to determine applicability of these interventions in our setting. If successful, these approaches have the potential for widespread application in geriatric healthcare services, will reduce the projected escalation of falls and fall-related injuries, and improve the quality of life of our older community.

**Trial registration:**

ISRCTN11674947

## Background

According to the United Nations’ worldwide statistics for the year 2009, the percentage of individuals aged 60 years and over will increase from 11% to 22% by 2050 [1]. In comparison to global numbers, Malaysia is expected to have an ageing society by 2035, where the population size of people aged 60 years and over will reach 44.11 million, 15% of the total population size [2]. Falls as a cause of death in Malaysia is ranked 150 in the world, compared to 80 in the United States of America and 4 (critical) in Thailand [3]. In developed countries, approximately 32% older people will fall within one year, where 20% of fallers will need medical attention, and less than one in ten cases result in a fracture [4-6]. Growth of the older population is expected to be associated with an increase in number fallers presented to health care services, which will incur higher health care and social care costs, unless effective interventions can be implemented widely.

A fall has been defined by the Prevention of Falls Network Europe Consensus as, “an unexpected event in which the participants come to rest on the ground, floor, or lower level” [7]. This broad definition has been adopted for the present study from the Kellogg’s International Working Group’s report in 1987, as our study will include falls that have occurred as a result of dizziness, syncope and cardiovascular abnormalities [8,9].

Published data on falls in older people from Malaysia is scarce. One Malaysian study suggested that injuries sustained from falls in older people are highly prevalent, constituting 40-60% of falls cases [10]. Previous studies have also identified the occurrence of ‘post-fall syndrome’ in fallers. Individuals who have a fear of falling appear to enter a debilitating spiral of loss of confidence, functional decline and social deprivation, physical frailty, falls, and loss of independence [11,12]. Hypothermia, pressure-related injuries and infections may also result from falls [13].

Falls is not a part of the natural ageing process. They are often the result of a collective and individualized set of risk factors [9,14]. The total count of possible risk factors contributing to falls exceeds 400, among the many are impaired sensorimotor functioning, cardiovascular complications, muscle weakness, environmental factors, osteoarthritis, visual limitations and depression [15,16]. Falls are multifactorial, the culmination of various combinations of intrinsic and extrinsic factors [6]. Thus, assessment of fallers needs to be multifaceted, to identify potentially modifiable risk factors that could be addressed in order to reduce the overall falls risk. A recent Cochrane review has identified that multifactorial interventions are effective in reducing falls [5].

Majority of randomized controlled trials in falls prevention were focused in developed, Western nations. In developing countries like Malaysia, the concept of ‘geriatrics’ is still at its infancy. As a result, there are few structured geriatric support services. Falls research is also under developed in Malaysia. Falls risk factors have been shown to be dependent on cultural differences and living conditions of the elderly [17]. So, interventional approaches previously shown to be effective in developed countries may not be effective in a developing community setting. A recent Cochrane review concluded that “these are complex interventions, and their effectiveness may be dependent on factors yet to be determined” [5]. Therefore, this study will be among the first few multifactorial falls intervention studies in an Asian community. Our main objective is to evaluate the effectiveness of an individually-tailored multifactorial intervention in reducing falls among the at risk older population in Malaysia.

## Methods/Design

### Trial design

**Figure 1:**
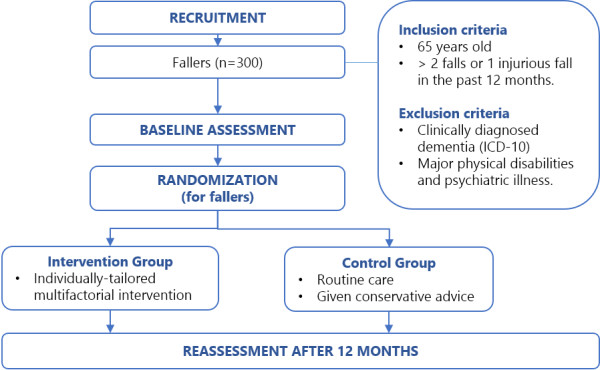
The Malaysian Falls and Intervention Trial (MyFAIT) flowchart.

This study is a pragmatic single-blind (assessor) randomized controlled trial of an individually-tailored multifactorial intervention for older Malaysian fallers. The study will be aptly named the Malaysian Falls Assessment and Intervention Trial (MyFAIT). The CONSORT statement is used as a guideline to design the flow of participants’ progress through the study (see attached Figure 1).

### Ethics approval

This study has received ethics committee approval from the University of Malaya Medical Ethics Committee. All potential participants will be provided with a plain language statement, and a detailed explanation of what participation in the study involves. Written consent will be taken from all individuals who agree to be involved in the study.

### Participant recruitment and randomization

The inclusion criteria will consist of participants who satisfy two criteria: (i) being aged 65 years or older with (ii) a history of two or more falls, or one injurious fall in the past 12 months. Injuries will be classified according to the International Classification of Diseases, 10th Edition (ICD-10) [7]. Participants will be excluded if they have at least one of the following: (i) clinically diagnosed dementia (ICD-10 definition), (ii) severe physical disabilities (i.e. unable to walk with a walking aid), or (iii) major psychiatric illnesses, psychosis (i.e. schizophrenia, paranoia) or brain damage. The study will be conducted in University Malaya Medical Centre (UMMC), Kuala Lumpur, Malaysia. Three hundred urban-dwelling older adult fallers will be recruited from the primary care unit, geriatric clinic, the accident & emergency department and through referral from other specialties.

### Baseline assessment

All participants will receive a baseline assessment in the hospital’s geriatric facilities, prior to randomization (i.e. assessment will be blind to group allocation). Assessors will be by the research team consisting of geriatricians, psychiatrists, ophthalmologist, physiotherapist and researchers who have received training in administration of all assessment items. Data will be collected on falls history, comorbidities, medication history, functional ability, psychological and socio-demographic status. On average, the baseline assessment is expected to last 2 hours per participant. Nutritional health data will not be collected.

#### Physical strength and balance performance

##### Hand-grip strength

This is measured using a Jamar dynamometer with the participant seated upright and elbow flexed at 90°. Measurements will be repeated three times in each hand, and the average of the three trials will be used for analysis. Grip strength is a key prognostic indicator of clinical and functional performance caused by underlying diseases [18,19].

##### Functional reach

The participant will be asked to stand with feet together, with the dominant arm positioned in 90° flexion. The participant will be asked to reach forwards as far as they can without overbalancing. The maximum distance reached is recorded. Shoes will be kept on for this test. Functional Reach has been shown to be able to detect balance disorders and identify change in balance performance over time [20].

##### Timed Up-and-Go (TUG)

The is the time taken for participants to stand from a 46 cm-high chair with arms, walk forwards 3 meters at regular walking pace, turn back, and return to the original sitting position with their back against the back of the chair. This will be recorded in seconds. Shoes will be kept on for this test. Participants will be allowed to use their usual indoor/outdoor walking aid to perform this test. A meta-analysis of TUG has shown this test to have moderate discriminative capacity in distinguishing fallers from the non-fallers [21,22].

#### Visual assessment

(i) Visual acuity, (ii) Contrast sensitivity, and (iii) Binocular vision test will be conducted using standardized methods utilizing the Snellen chart, Pelli-Robson charts and Frisby Stereotest (Near) respectively [23-25].

#### Baseline cardiovascular assessment

A baseline resting 12-lead electrocardiogram (ECG) will be recorded. Postural blood pressure will be recorded using non-invasive continuous blood pressure monitoring device (Portapres®, Finapres Medical Systems). Following 10 minutes of rest in a supine position, participants will be asked to stand for three minutes. Orthostatic hypotension will be diagnosed if a reduction in systolic blood pressure of ≥20 mmHg, or diastolic blood pressure of ≥10 mmHg within three minutes of standing is observed [26,27].

#### Psychological and quality-of-life assessments

These will be assessed using psychometric questionnaires: Stress, anxiety and depression will be assessed using the 21-item Depression Anxiety Stress Scale (DASS-21) [28], quality of life will be assessed using the CASP-19 [29], and fear of falling will be assessed using the 7-item short Falls Efficacy Scale-International (FES-I) [30]. All these questionnaires have items on 4-point Likert scales, and have been shown to be reliable and valid in older populations [31-33].

### Randomization

The 300 fallers will be randomized to either the intervention or the control group. A computer-generated random number sequence will be created by an independent investigator. Treatment allocation will be concealed in sealed, opaque envelopes and stored in a secure location. To prevent bias, 1 year follow-up assessors will be blind to group allocation. And data analysis will be undertaken by a statistician who is blind to group allocation.

#### Control group

The control group will be provided with health advice and will continue to receive usual care by their medical practitioner and other health professionals.

#### Intervention group

Participants randomized to the intervention group will be prescribed individualized treatment programs by a medical specialist. The prescribed treatment will target six specific treatment modalities: falls education, exercise intervention, home hazards intervention, cardiovascular intervention, visual intervention, and medication review. All participants of the intervention arm will receive falls education. The decision for the Otago exercises, home hazards assessment and visual intervention will be made according to predefined criteria. Decisions for detailed cardiovascular evaluation and medication review will be based on clinical decisions made according to specific features in the participants’ clinical and medical history.

##### Falls education

An information leaflet will be sent to all individuals randomized to the intervention group. The leaflet will contain information about common risk factors and simple falls prevention strategies. It serves to increase awareness, and as a reinforcement tool on the risk factors for falls and the potential benefits of various interventions, based on the findings of previous research.

##### Otago exercise program

Participants will be referred for physiotherapy if their Timed Up-and-Go (TUG) score is 13.5 seconds or above [21]; or if they have a clinically apparent gait and balance disorder. An individually-tailored home exercise program will be prescribed based on a modified Otago exercise program, which has been shown to be highly effective in reducing injurious fall rates among those who have a previous history of fall [34]. The program focuses on balance and strengthening exercises of 5–8 hospital exercise sessions, with recommended home practice of at least 5 times a week. The participant will be invited to attend the hospital exercise sessions every month for three months where the intensity of the exercises will be increased as appropriate, and also to motivate ongoing participation. This is followed by recommendations for engagement in other regular physical exercises like Tai Chi, which have been shown to reduce falls [35].

##### Home environment modifications

Participants will be referred for occupational therapy if their fall occurred at home and is non-syncopal in nature. The Home Falls and Accident Screening Tool (HOME FAST) will be administered by a trained occupational therapist to systematically detect home environmental hazards and activities in the home that could potentially increase an individual’s risk of falling [36]. Appropriate modifications will then be recommended. To facilitate immediate implementation, equipment will be provided and installed upon completion of environmental assessment with the home owner’s consent. A second home visit will be conducted to review and encourage usage of modifications and equipment.

##### Cardiovascular intervention

Participants with a history of unexplained falls or falls associated with symptoms of dizziness, presyncope or syncope will be further investigated with cardiovascular investigations. These will include ambulatory ECG monitoring, transthoracic echocardiography, carotid sinus massage or head-up tilt-table tests or cardiac stress tests according to the history provided [37]. If hypotensive disorders of orthostatic hypotension, vasovagal syncope or vasodepressor carotid sinus hypersensitivity are confirmed, participants will be treated with conservative advice, withdrawal of culprit medication and pharmacological interventions with vasopressors according to clinical severity. A referral for permanent pacemaker insertion or other cardiac interventions will be provided if necessary [38].

##### Visual interventions

For individuals with a visual acuity of 6/12 or worse in either eye, the participant will be referred to the hospital’s ophthalmologist for further assessment. These will also include additional assessments for refraction errors, cataracts, retinal disorders and glaucoma. Interventions such as prescription or revision of eye glasses, cataract surgery and glaucoma treatment will be prescribed according to clinical indication.

##### Withdrawal and review of culprit medications

Following a detailed medication history, falls-risk increasing drugs (FRID) in older adults will be reviewed and withdrawn if needed. The withdrawal of potential culprit medications will be performed by the study’s geriatrician [39].

### Outcome assessment

The primary outcome measure is the proportion of fallers between the intervention and control groups, while the secondary outcome measure is the rate of falls between the two groups.

Falls diaries with daily entries will be used to record falls occurrences, and will be returned monthly for one year from randomization, as recommended by previous studies [7]. These diaries are written in the three main languages of Malaysia; Malay, English and Mandarin, due to varying cultural and educational backgrounds. Prompts will be provided in the falls diaries, which include how and when a fall occurred, or if there were injuries sustained after their fall. Participants will be contacted every 2 months by telephone calls to encourage complete diary returns. The participant will be followed-up for one year, and then invited back for a re-assessment using the same baseline parameters. Adherence to interventions will be recorded for sub-analysis.

Additional secondary outcomes will include changes in ECG, postural blood pressure, visual, physical and psychometric assessments from the baseline assessment to one year follow-up.

### Statistical analysis and power calculation

One hundred and thirty three participants per group (n = 266) will provide an 80% power (α = 0.05) to detect a 40% reduction in the number of individuals who experience falls, assuming that without intervention, 50% of individuals will experience a subsequent fall (based on previous studies of high risk populations) [40,41], and a 30% drop out rate (accounting for a likely high mortality and morbidity in our participants).

All analysis will be performed on an intention-to-treat basis with the exception of those who have withdrawn or died. Missing data imputations will be performed on variables with high probability of missingness, either by discarding or replacing data with the mean of the observed values for that variable. Fall and injury rates will be assessed using negative binomial model and multiple regression. For secondary outcomes, comparisons will be conducted between the intervention and control groups. Changes within groups from baseline and potential covariates will be examined with multiple regression and paired t-test for normally-distributed continuous data and Kruskall-Wallis for skewed continuous data.

## Discussion

Multifaceted interventions in previous studies have demonstrated the effectiveness of this approach in reducing falls compared to single or multicomponent interventions [42]. However, a meta-analysis of multifaceted interventions by the most recent Cochrane review has found that although it reduces the number of falls, multifactorial interventions do not help reduce the risk of falling [5]. Strong positive outcomes were observed especially in studies with land-based exercise, medication withdrawal, vitamin D supplementation, footwear intervention, and home environment modification [14,43].

There is however insufficient evidence that multifactorial interventions can be conducted outside a tightly controlled group of research trials and implemented into clinical practice and community services [42]. There is also a lack of evidence to prove the effectiveness of visual intervention, medications withdrawal and falls prevention education in reducing the number of falls [14,39]. Most studies were primarily performed in Western countries, and very few RCT’s have been conducted in low and middle-income developing countries, which will be achieving the ‘ageing population’ status in a few decades, at a substantially faster rate than the Western nations [2,44,45]. Although many falls-related trials have been conducted, interventions or outcomes were rarely implemented in a large-scale clinical setting. Edwards theorized that the baton for falls prevention needs to be passed on by falls experts to medical practitioners for it to be incorporated into common clinical practice. Active participation of the government, policymakers and research funders is also vital [45,46].

It is also important to take note of ethnicity and also where older fallers are recruited from. Fallers from the Stroke Unit or Accident and Emergency Department are characteristically more dependent, prone to recurring falls, and are more receptive to interventions [4,46-48]. Lee and colleagues reported 25.2% of 1-year falls incidence when recruiting fallers from medical and community health centers [4]. A systematic review of worldwide Chinese fallers reported a lower rate of 18% [49]. The frequently reported incidence rate of 30% annual falls in elderly [8], may therefore fluctuate depending on faller characteristics.

Cultural diversity may play an important role in falls interventions. Chinese older people residing in London were reluctant to talk about falls, tending to hide falls from their adult children to avoid worrying them; and have poor knowledge about the availability and content of interventions [13,49]. The results of Western studies should not be directly extrapolated to Malaysia and other Asian countries due to demographic, lifestyle, cultural, environmental and health service system differences. Little is currently known about how falls can be appropriately managed in developing countries. In our setting where the population is ageing at a far faster rate than the Western developed countries, cost-effective solutions to age-related conditions are desperately needed. Hence it is important to evaluate the effectiveness of the falls intervention program on local fallers before developing relevant falls interventions in clinical practice.

Using a pragmatic RCT approach to falls, MyFAIT will evaluate the effectiveness of a multifactorial intervention in reducing the incidence of falls, and the rate of falling as a secondary prevention measure in older fallers seeking medical attention in primary and secondary care settings. This project will fill a vital gap in global falls prevention measures by targeting a culturally-diverse older Asian community of low-middle income. This study will also help to determine the characteristics of fallers in our setting and detect potential differences or similarities between existing falls research populations. If successful, these approaches have the potential for widespread application in geriatric healthcare services and should minimize the projected escalation of falls related health service needs in the future, and improve the quality of life of our older community.

## Conclusion

While multifactorial falls interventions have been demonstrated to be effective in reducing frequency of falls in Western populations and used in many falls intervention program in developed countries, such an intervention has not been adequately evaluated in lower income countries in Asia. We are conducting a randomized-controlled study in a middle-income developing country in South-East Asia. The results of this study will provide much needed information about the potential benefits of multifactorial falls intervention outside the developed countries. This study will also provide valuable information on the characteristics of fallers and the response to individual interventions within the multifactorial program.

## Abbreviations

WHO: World Health Organization; MyFAIT: Malaysian Falls Assessment and Intervention Trial; ICD-10: International Classification of Diseases, 10th Edition; UMMC: University Malaya Medical Centre; TUG: Timed Up-and-Go; ECG: Electrocardiogram; DASS-21: Depression Anxiety Stress Scale; FES-I: Falls Efficacy Scale-International; HOME FAST: Home Falls and Accident Screening Tool; FRID: Falls-risk increasing drugs.

## Competing interests

This study is funded by the Health and Translational Medicine Cluster (HTMC) under the University of Malaya Research Grant (UMRG) program, and also by the Ministry of Science, Technology and Innovation (MOSTI) under the ScienceFund grant.

## Authors’ contributions

All authors have made an intellectual contribution to this research trial. MPT, EMK, PJHP and KH were responsible for identifying the research questions and design of the study and overseeing the implementation of the study. KC was responsible for the development of additional research questions, further consolidation of research design and statistical information. PJT contributed to the development of support materials, recruitment of participants and study implementation. All authors were responsible for drafting of this manuscript and have read and approved the final version.

## Authors’ information

Tan, Pey June, Khoo, Ee Ming, Chinna, Karuthan, Hill, Keith D., Poi, Phillip J. H., Tan, Maw Pin are co-authors.

## Pre-publication history

The pre-publication history for this paper can be accessed here:

http://www.biomedcentral.com/1471-2318/14/78/prepub
